# Stemness Potency of Human Gingival Cells—Application in Anticancer Therapies and Clinical Trials

**DOI:** 10.3390/cells9081916

**Published:** 2020-08-18

**Authors:** Katarzyna Stefańska, Katarzyna Mehr, Maria Wieczorkiewicz, Magdalena Kulus, Ana Angelova Volponi, Jamil A. Shibli, Paul Mozdziak, Mariusz T. Skowroński, Paweł Antosik, Jędrzej M. Jaśkowski, Hanna Piotrowska-Kempisty, Bartosz Kempisty, Marta Dyszkiewicz-Konwińska

**Affiliations:** 1 Department of Histology and Embryology, Poznan University of Medical Sciences, 6 Święcickiego St., 60-781 Poznan, Poland; k.stefanska94@o2.pl; 2 Department of Gerostomatology and Pathology of Oral Cavity, Poznan University of Medical Sciences, 70 Bukowska St., 60-812 Poznan, Poland; katarzynamehr@gmail.com; 3 Department of Basic and Preclinical Sciences, Institute of Veterinary Medicine, Nicolaus Copernicus University in Torun, 7 Gagarina St., 87-100 Torun, Poland; maria.wieczorkiewicz@umk.pl (M.W.); skowron@umk.pl (M.T.S.); 4 Department of Veterinary Surgery, Institute of Veterinary Medicine, Nicolaus Copernicus University in Torun, 7 Gagarina St., 87-100 Torun, Poland; magdalena.kulus@umk.pl (M.K.); pantosik@umk.pl (P.A.); 5 Centre for Craniofacial and Regenerative Biology, Dental Institute, King’s College London, Strand, London WC2R 2LS, UK; ana.angelova@kcl.ac.uk; 6 Department of Periodontology and Oral Implantology, Dental Research Division, Guarulhos University, Guarulhos, R. Eng. Prestes Maia, 88-Centro, São Paulo 07023-070, Brazil; jashibli@yahoo.com; 7 Physiology Graduate Program, North Carolina State University, Campus Box 7608, Raleigh, NC 27695-7608, USA; pemozdzi@ncsu.edu; 8 Department of Diagnostics and Clinical Sciences, Institute of Veterinary Medicine, Nicolaus Copernicus University in Torun, 7 Gagarina St., 87-100 Torun, Poland; jmjaskowski@umk.pl; 9 Department of Toxicology, Poznan University of Medical Sciences, 30 Dojazd St., 60-631 Poznan, Poland; hpiotrow@ump.edu.pl; 10 Department of Anatomy, Poznan University of Medical Sciences, 6 Święcickiego St., 60-781 Poznan, Poland; m.dyszkiewicz@ump.edu.pl; 11 Department of Biomaterials and Experimental Dentistry, Poznan University of Medical Sciences, 70 Bukowska St., 60-812 Poznan, Poland

**Keywords:** gingiva, gingival mesenchymal stem cells, mesenchymal stem cells, mesenchymal stromal cells, cancer, exosomes

## Abstract

Gingivae, as the part of periodontium, are involved in tooth support and possess the ability to heal rapidly, without scar formation. Recently, dental tissues have been identified as a potential source of mesenchymal stem cells (MSCs) and several populations of MSCs were isolated from the orofacial region, including gingival mesenchymal stem cells (GMSCs). GMSCs exhibit robust immunomodulatory and differentiation potential and are easily obtainable, which make them promising candidates for cellular therapies. Apart from being tested for application in immunologic- and inflammatory-related disorders and various tissue regeneration, GMSCs promise to be a valuable tool in cancer treatment, especially in tongue squamous cell carcinoma (TSCC) with the use of targeted therapy, since GMSCs are able to selectively migrate towards the cancerous cells both in vitro and in vivo. In addition to their ability to uptake and release anti-neoplastic drugs, GMSCs may be transduced with apoptosis-inducing factors and used for cancer growth inhibition. Moreover, GMSCs, as most mammalian cells, secrete exosomes, which are a subset of extracellular vesicles with a diameter of 40–160 nm, containing DNA, RNA, lipids, metabolites, and proteins. Such GMSCs-derived exosomes may be useful therapeutic tool in cell-free therapy, as well as their culture medium. GMSCs exhibit molecular and stem-cell properties that make them well suited in preclinical and clinical studies.

## 1. Introduction

Mesenchymal stem cells (MSCs) comprise a heterogenous subset of stromal cells that have fibroblast-like morphology, form colonies in vitro, and proliferate as plastic-adherent cells [1]. MSCs were isolated for the first time by Friedenstein et al. [2] from the bone marrow, defined as colony-forming unit fibroblasts (CFU-Fs) and were demonstrated to possess self-renewal abilities. Subsequently, their multilineage differentiation potential was reported [3], suggesting that MSCs may be utilized in cellular therapies and regenerative medicine. Since then, MSCs have been isolated from various tissues, such as adipose tissue [4], placenta [5], amniotic fluid [6], fetal liver [7], or umbilical cord [8]. Although embryonic stem cells (ESCs) are pluripotent, they possess the ability to differentiate into all three primary germ layers, and their acquisition is ethically controversial, since they are derived from the inner cell mass of the preimplantation blastocyst [9]. MSCs utilization is not burdened with such concerns, as they can be obtained from adult tissues.

The orofacial region gained lot of interest as a potential source of MSCs as well. To date, eight distinct populations of dental-derived MSCs have been obtained, with dental pulp stem cells (DPSCs) being isolated as first by Gronthos et al. [10]. Subsequent studies led to isolation of stem cells from human exfoliated deciduous teeth (SHED) [11], periodontal ligament stem cells (PDLSCs) [12], dental follicle progenitor cells (DFPCs) [13], alveolar bone marrow stromal cells (ABMSCs) [14], stem cells from the apical papilla (SCAP) [15], tooth germ progenitor cells (TGPCs) [16], and gingival mesenchymal stem cells (GMSCs) [17].

The gingiva, as the majority of the periodontal tissues, arises from the neural crest ectomesenchymal origin (N-GMSCs); however, Xu et al. indicated that about 10% of GMSCs is derived from mesoderm (M-GMSCs) [18]. Compared to M-GMSCs, N-GMSCs preferably differentiate into neural cells, accompanied by an increase in nestin, neurofilament M (NF-09), and β-tubulin III expression and the elevated expression of Fas ligand (FasL). However, both subpopulations show no difference in osteogenic and adipogenic differentiation. In terms of histological structure, the gingiva consists of connective tissue and epithelium and, as a part of the periodontium, is involved in tooth support, surrounding the tooth and being attached to the alveolar bone, forming the gingival attachment [19]. The gingival tissue seems to be a particularly attractive source of stem cells, given its fast regeneration after injury without scar formation, compared to the skin healing abilities, and the fact that obtaining it is minimally invasive for the patient [20]. Moreover, it may be used in autologous transplant, with no need for search for a matching donor.

Mesenchymal stem cells have been a major focus of regenerative medicine in recent years. They offer a promise to manage diseases such as rheumatoid arthritis, systemic lupus erythematosus, multiple sclerosis, diabetes mellitus, neurological disorders, and many others [21]. Thus, there is constant ongoing search for easily available MSCs that could be applied in clinical environment, and GMSCs seem to fulfill those requirements. Therefore, the objective of this review is to describe the methods of isolation and cultivation of human gingival cells, their molecular properties and plasticity, and possible clinical application.

## 2. Methods of GMSCs Isolation and Cultivation

For GMSCs isolation, gingival tissue samples are obtained during standard dental procedures, during which they constitute biological waste and are then used for research, or as a targeted procedure, e.g., as a “gingiva punch.” Such tissue is then deprived of epithelium and the remaining connective tissue is either minced and enzymatically digested or cut into smaller pieces, in the explant method. In case of the explant method, the gingiva is minced with a sterile scalpel and such obtained tissue pieces are placed in tissue culture dishes, for plastic-adherent cells outgrowth. The basal media used for the culture varied amongst researchers, including Minimum Essential Medium α (α-MEM) [22,23] or Dulbecco’s Modified Eagle’s Medium (DMEM) [24,25]. In most cases, the media were supplemented with fetal bovine serum (FBS) of different concentrations [22,24]. However, some investigators utilized fetal calf serum (FCS) [23,25]. In a study by Fawzy El-Sayed et al. [23], the magnetic activated cell sorting (MACS) was used to obtain STRO-1 positive population of GMSCs after initial isolation. As a result, they obtained two distinctive populations of gingiva-derived cells; STRO-1/MACS+ cells were positive for mesenchymal stromal cell markers defined by ISCT [26] and exhibited multilineage differentiation potential and CFU (colony forming unit) capacity, whereas STRO-1/MACS− cell fraction expressed hematopoietic markers, such as CD34 or CD45, and multilineage differentiation potential was not evident, suggesting that this population of cells lack many of stem cell properties [23]. Importantly, Rao et al. [27] demonstrated, that STRO-1 expression in GMSCs decreased with an increasing passage number, suggesting that this marker may be useful for undifferentiated GMSCs isolation.

In case of the other aforementioned method, the tissue samples are diced and digested in enzymatic solutions of various compositions and then filtered through strainers to obtain single cell suspensions. The enzymes used by the researchers included collagenase IV [17,28], collagenase I [29], or a combination of various types of collagenase and Dispase [30,31,32]. Amongst the researchers using the enzymatic method, Ge et al. [31] attempted to isolate MSCs from inflamed gingival tissue, taking into consideration its abundance and accessibility. Ge et al. [31] discovered that such GMSCs exhibited proliferative potential and expressed markers characteristic for MSCs, therefore suggesting that inflamed gingiva may provide a valuable source of MSCs instead of being discarded as a biological waste [31]. In case of all aforementioned studies, the in vitro culture was conducted in a humidified tissue culture incubator with 5% CO_2_ at 37 °C. A more detailed overview of GMSCs isolation via enzymatic and explant method is provided in Table 1.

## 3. Stemness Properties and Differentiation Ability of GMSCs

GMSCs fulfill the minimal criteria to be considered as mesenchymal stem cells, according to the International Society for Cellular Therapy (ISCT) [26], meaning they are plastic-adherent in standard culture conditions, express specific surface antigens, which include CD105 (Endoglin), CD73, and, CD90 (Thy-1), do not express CD45, CD34, CD14, CD11b, CD79α, CD19, and HLA-II (human leukocyte antigen II), and are able to differentiate into osteoblasts, chondroblasts, and adipocytes [26]. Many investigators have decided to expand this list of markers, revealing that GMSCs also express CD29 (Integrin beta 1) [17,30], CD44 [22,25,30,32], CD146 [29], and even proteins considered as markers of pluripotency or embryonic stem cell markers, namely, Oct-4 (octamer-binding transcription factor 4), STRO-1, SSEA-4 (stage-specific embryonic antigen 4), and Nanog [33,34]. Primary cultures of GMSCs comprise uniformly homogenous population of spindle-shaped fibroblast-like cells, contrary to bone marrow-derived MSCs (BM-MSCs), which require two to three passages to become homogenous [30]. They are also more proliferative and genetically stable than BM-MSCs, show normal karyotype and are nontumorigenic in both early and late passages and expand independently on external growth factors [30].

Osteogenic differentiation was carried out in a number of studies by conducting GMSCs culture in osteoinductive medium with addition of dexamethasone, ascorbic acid, and β-glycerophosphate (among others) [22,25,28,29,32,35,36,37]. The results of differentiation were confirmed with Alizarin Red S staining to detect calcified deposits in culture, alkaline phosphatase (ALP) activity assay, and gene expression analysis of bone specific markers—Runx2 (Runt-related transcription factor 2), collagen I, ALP, osteocalcin (OCN), and bone sialoprotein (BSP) [22,25,28,29,32,35,36,37]. Moreover, GMSCs exhibited higher osteogenic potential than BM-MSCs, as indicated by Sun et al. [36].

In case of adipogenic differentiation, the culture medium was supplemented with 1-methyl-3-isobutylxanthine, indomethacin dexamethasone, insulin, and L-glutamine, and the presence of lipid droplets was evaluated with oil red O staining ([22,25] among others). Interestingly, the addition of muscone, one of the main components of natural musk, to the culture medium enhanced the adipogenic differentiation of GMSCs, whereas the osteogenic differentiation was decreased when the inhibition of the Wnt/β-catenin signaling pathway was present [38].

For chondrogenic differentiation, the culture medium was supplemented with different combinations of TGF-β, dexamethasone, ascorbic acid, insulin, bone morphogenetic protein 6, and insulin-transferrin selenium acid [22,25]. The results of differentiation were confirmed by safranin O staining for glycosaminoglycans [22] and toluidine blue, which highlights the acid proteoglycans [25].

Apart from this trilineage differentiation potential, the ability of GMSCs to transform into neurons and endothelial cells was demonstrated by Zhang et al. [17]. They reported that after 1 week culture in endothelial growth medium, GMSCs started to express CD31 (PECAM-1), which is a marker for endothelial cells. Importantly, under normal culture conditions, its expression was not observed. After the cultivation in neuroinductive conditions, GMSCs were positive for glial fibrillary acidic protein (GFAP), neurofilament 160/200 (NF-M), and β-tubulin III [17]. On the contrary, Marynka-Kalmani et al. [39] reported a decrease in β-tubulin III and glial fibrillary acidic protein expression and induction of NeuN and MAP2 (microtubule associated protein 2) expression. In the same study, GMSCs subjected to glial differentiation regimen were shown to induce neuritogenesis and support PC12 cells survival [39]. Neurogenic differentiation of GMSCs could be enhanced by low-intensity pulsed ultrasound (LIPUS) [24], hypoxia preconditioning [40] or treatment with cannabidiol (CBD) [41]. Rao et al. [42], on the other hand, successfully differentiated GMSCs encapsulated in the bioconjugated protein hydrogel (3D) into neuronal lineage, which was confirmed by presence of Nissl bodies, as well as the elevated expression of nestin, β-tubulin III, and MAP2.

**Figure 1:**
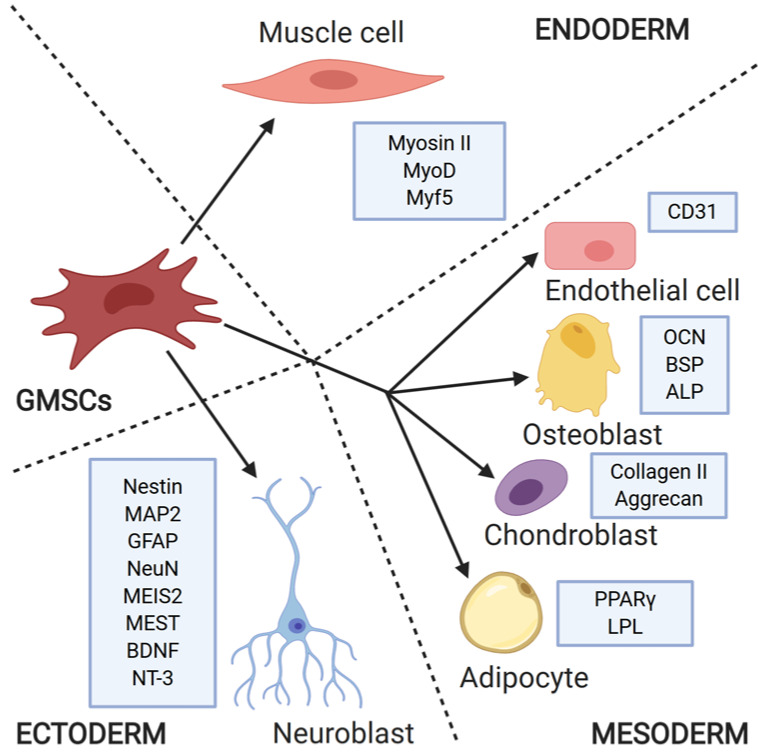
GMSCs were differentiated into lineages derived from all three primary germ layers, which was confirmed with the presence of cell-specific markers. Abbreviations: MyoD—myoblast determination protein 1; Myf5—myogenic factor 5; OCN—osteocalcin, BSP—bone sialoprotein; ALP—alkaline phosphatase; PPARγ—peroxisome proliferator-activated receptor γ; LPL—lipoprotein lipase; MAP2—microtubule associated protein 2; GFAP—glial fibrillary acidic protein; NeuN—neuronal nuclei; MEST—mesoderm-specific transcript homolog protein; BDNF—brain-derived neurotrophic factor; NT-3—neurotrophin 3. Created with BioRender.

To accomplish myogenic differentiation, GMSCs were encapsulated in alginate microspheres containing growth factors and cultured in medium with the addition of dexamethasone, ascorbic acid, sodium pyruvate, forskolin, and bFGF [43]. After 2 weeks of in vitro cultivation, the morphology of GMSCs changed towards myogenic cells; after 4 weeks, immunofluorescence staining revealed presence of myogenic markers, such as Myosin II (MF20), MyoD, and Myf5, which was confirmed by subsequent gene expression analysis [43]. The differentiation potential of GMSCs is presented in Figure 1.

## 4. Immunomodulatory Properties

Apart from their self-renewal and differentiation capacities, MSCs also exhibit a broad range of immunomodulatory properties, which may be exerted by direct cell-to-cell contact or in paracrine manner, via soluble factors production (e.g., IL-1 (interleukin 1), IL-6, IL-10, indoleamine 2,3-dioxygenase (IDO), nitric oxide (NO), transforming growth factor β1 (TGFβ1), and prostaglandin E2 (PGE2)) [44]. Importantly, such immunoregulation may occur in various ways, depending on the level of inflammation in the surrounding environment, as indicated by Li et al. [45]. When activated T cells produced low levels of inflammatory IFNγ (interferon γ) and TNFα (tumor necrosis factor α), MSCs enhanced their proliferation, supporting the immune reaction. On the contrary, the higher amount of these cytokines enhanced immunosuppressive properties of MSCs [45].

Zhang et al. [17] reported that GMSCs inhibited peripheral mononuclear blood cell (PBMC) proliferation and cytokine secretion in response to mitogen stimulation. Their results suggested that IDO (that is not constitutively expressed by GMSCs) and IL-10, but not TGFβ1, COX2 or iNOS, were involved in this immunosuppression. Moreover, the secretion of IDO and IL-10 by GMSCs was stimulated by IFNγ [17]. Similar results were obtained by Davies et al. [46] in oral mucosal progenitor cells, who also reported IDO upregulation in these cells in response to IFNγ stimulation. Moreover, Jiang et al. [47] indicated that hypoxia enhanced IL-10 production and FasL (Fas ligand) expression in GMSCs, which resulted in increased PBMCs apoptosis. The mechanism of FasL/Fas-mediated T cell apoptosis inflicted by MSCs (BM-MSCs, specifically) was described by Akiyama et al. [48], and FasL expression in GMSCs derived of neural crest ectomesenchyme was also reported by Xu et al. [18].

**Figure 2:**
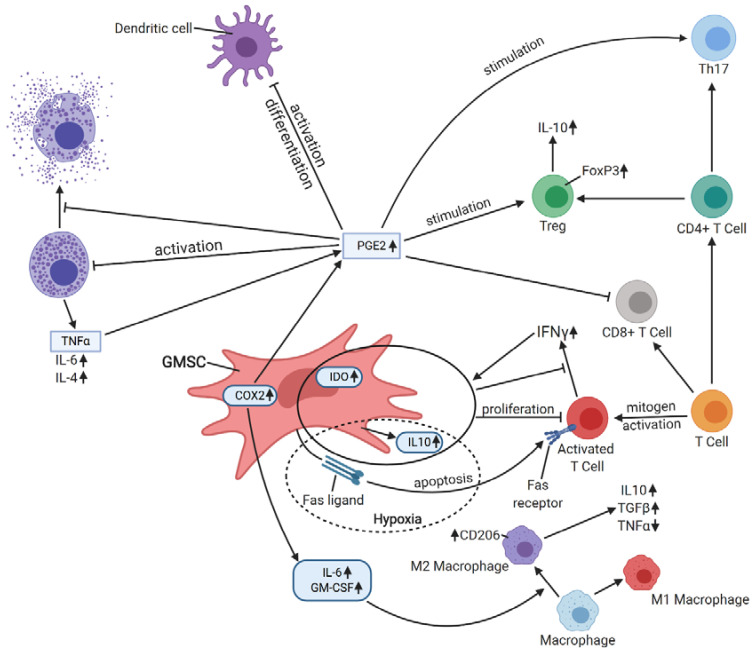
A schematic immunomodulatory activity of GMSCs. GMSCs exert apoptotic effects on T-cells via FasL/Fas pathway, and FasL expression and IL-10 secretion by GMSCs is enhanced by hypoxia. In response to IFNγ produced by activated T cells, GMSCs secrete IDO and IL-10, which inhibit T cells’ proliferation and IFNγ secretion. GMSCs elicit M2 polarization of macrophages via secretion of IL-6 and GM-CSF, which results in an increased expression of CD206, higher secretion of IL-6 and TGFβ and reduced production of TNFα by M2 macrophages. The activation and differentiation of dendritic cells is attenuated via PGE2-dependent mechanism, as well as the activation and degranulation of mast cells. Additionally, TNFα secreted by mast cells increases the production of PGE2 in GMSCs. PGE2 is also responsible for CD8+ T cells’ inhibition and Treg and Th17 cells’ stimulation. Abbreviations: IDO—indoleamine 2,3-dioxygenase, IL-10—interleukin 10, IL-6—interleukin 6, IL-4—interleukin 4, FasL—Fas ligand, GM-CSF—granulocyte-macrophage colony-stimulating factor, COX2—cyclooxygenase 2, PGE2—prostaglandin E2, IFNγ—interferon γ, TGFβ—transforming growth factor β, TNFα—tumor necrosis factor α, FOXP3—forkhead box P3. Created with BioRender.

Su et al. [49] reported using GMSCs in managing hapten-induced murine contact hypersensitivity (CHS) and observed a reduced infiltration of dendritic cells, CD8+ T cells, and Th17 effector cells in allergic areas, while infiltration of Tregs was enhanced. A number of mast cells, which are critical in allergic and inflammatory disorders [50], as well as the percentage of degranulated mast cells, were also decreased in response to the treatment. Such immunosuppressive effect on dendritic and mast cells was ascribed to PGE_2_, but not IL-10, TGFβ1, or IDO, production [49]. GMSCs were also demonstrated to elicit M2 polarization of macrophages and enhance cutaneous wound healing, which was due to the secretion of soluble factors by GMSCs, namely, IL-6 and GM-CSF [51]. After coculture with GMSCs, macrophages exhibited an increased expression of mannose receptor (CD206) and secreted IL-6 and IL-10. Moreover, their ability to induce Th17 cell expansion was reduced, as well as their production of TNFα, which was hypothesized to be due to impaired activation of NFκB p50 [51]. Zhao et al. [52] investigated the influence of GMSCs on T cell-mediated bone marrow failure in a mouse model. GMSCs reduced infiltration of CD8+ T cells, Th1 and Th17 cells, while differentiation of CD4+FOXP3+ Tregs was enhanced in lymph nodes. In addition, the serum levels of inflammatory cytokines, such as TNFα, IFNγ, or IL-6, decreased with an increase of anti-inflammatory IL-10, indicating that bone marrow failure was attenuated [52]. The schematic immunomodulatory activity of GMSCs is presented in Figure 2.

## 5. Preclinical Studies with GMSCs

The number of promising studies with GMSCs was conducted on animal models in tissue regeneration, inflammatory- and immunologic-related disorders, and tongue squamous cell carcinoma, among others. Importantly, besides using GMSCs themselves, their conditioned media were also proven to exert therapeutic properties. In some of the studies, GMSCs were engineered using advanced techniques, such as encapsulation in alginate microspheres, seeding on various types of scaffolds or 3D bioprinting.

### 5.1. Anticancer Therapies

Mesenchymal stem cells have been shown to preferentially migrate towards tumors, possibly due to the enhanced inflammation in the tumor microenvironment, making them a promising tool in anticancer therapies [53]. However, there are conflicting reports in the literature about the potential role of MSC in tumor microenvironment. Some authors reported tumoricidal effects of MSCs, e.g., on lung cancer cells [54], whereas in case of a breast cancer, MSCs promoted cancer progression and metastasis [55]. Promoting of tumor growth and angiogenesis may be exerted via secretion of proangiogenic cytokines, such as IL-6, VEGF (vascular endothelial growth factor), or TGFβ, by MSCs differentiated into cancer-associated fibroblasts [56,57]. MSCs were also reported to increase the level of lysyl oxidase, resulting in enhanced breast cancer metastasis [58]. Additionally, the vast immunomodulatory properties of MSCs may not necessarily be beneficial in terms of cancer. Patel et al. [59] reported that MSCs may protect breast cancer cell from immune clearance via inhibition of NK (natural killer) cells and cytotoxic T lymphocytes, while increasing the level of Treg cells. When it comes to the anticancer effect, it mainly indicates the mechanism of immune response modulation, regulation of cellular signaling, and apoptosis induction. BM-MSCs were reported to reduce colorectal cancer initiation and progression via reprogramming the macrophages to become regulatory cells associated with phagocytosis, lowering the inflammation level by decreasing local IL-6 concentration and decreasing Th17 cell activity [60]. Adipose tissue-derived MSCs, on the other hand, were shown to suppress growth of tumor cells due to IFNβ secretion [61]. The reason of such discrepancies in terms of MSCs influence on cancer is probably due to the fact that MSCs from different sources may exert various effects on cancer cells. Ji et al. [62] suggested that MSCs for anticancer therapy should be derived from the same tissue/organ origin as tumor cells and that they would restore altered homeostasis, exerting anticancer effect.

To date, only a few studies utilizing GMSCs in anticancer therapies have been published (summarized in Table 2), mostly focusing on oral carcinomas, mainly tongue squamous cell carcinoma (TSCC). Squamous cell carcinomas comprise 95% of all head and neck carcinomas, whereas oral squamous cell carcinomas constitute more than 90% of oral neoplasms [63]. Traditional therapy for squamous cell carcinomas includes surgical resection and postoperative radiation. Although the constant development occurs in traditional anticancer therapies, the 5-year survival rate for this type of cancer is only 50%. Moreover, up to 30% of patients with TSCC exhibit metastatic lesions in the lymph nodes, even without the clinical signs of metastasis [64], clearly suggesting that another approaches must be undertaken to enhance the efficiency of cancer treatment. Ji et al. [62] cocultured GMSCs with two oral cancer cell lines—CAL27 and WSU-HN6, revealing that MSCs inhibited oral cancer cell growth in vitro. Moreover, their conditioned medium exerted the same effect, suggesting that soluble factors secreted by GMSCs (including IL-6, IL-8, and GM-CSF) may be responsible for such antiproliferative properties. Additionally, Western blotting revealed that GMSCs downregulated the proliferation-related gene expression in cancer cells (Bcl-2 and survivin), while upregulating apoptosis-related genes (Bax, cleaved caspase-3, and PARP). It was also suggested that GMSCs’ conditioned medium deactivated the JNK, ERK, and STAT3 pathways associated with proliferation [62].

**Figure 3:**
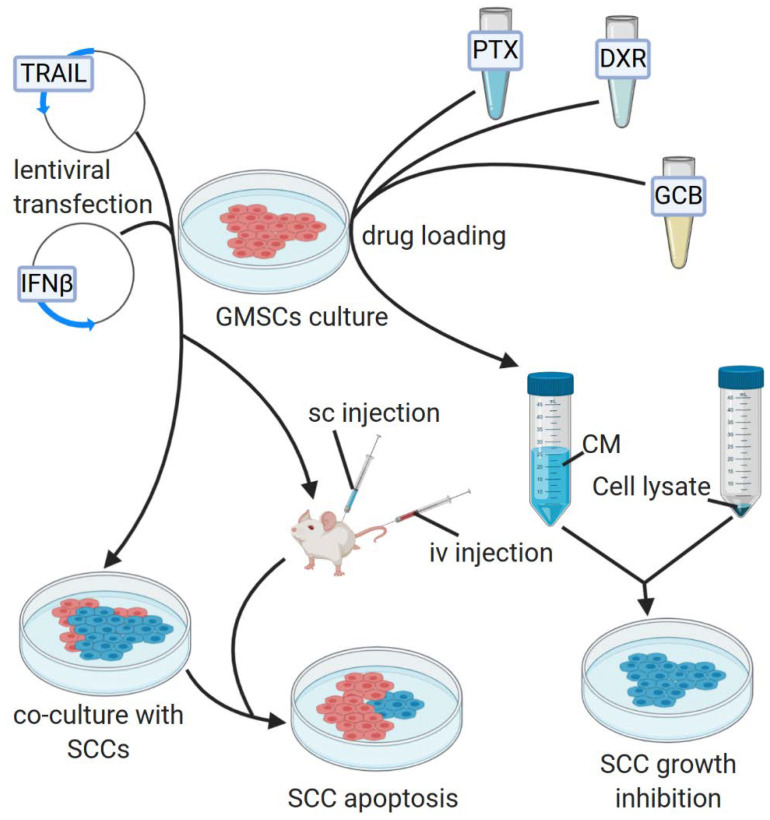
Overview of GMSCs engineering for enhanced anticancer properties. GMSCs may be transfected with lentiviral vectors, containing anticancer genes or loaded with anticancer drugs. Abbreviations: TRAIL—tumor necrosis factor-related apoptosis-inducing ligand; IFNβ—interferon β; SCC—squamous carcinoma cell; CM—culture medium; sc—subcutaneous; iv—intravenous; PTX—paclitaxel; DXR—doxorubicin; GCM—gemcitabine. Created with BioRender.

Engineering MSCs to express specific antitumor factors seems to be an interesting approach in anticancer therapies, because it could ensure sufficient level of given factor at the tumor site due to its long-term expression, which would not be possible via systemic administration. Methods of engineering GMSCs for their enhanced anticancer properties are presented in the Figure 3. Xia et al. [63] demonstrated that GMSCs migrate towards TSCC cell lines in a greater deal than towards 293T cells or culture medium. Therefore, they transduced GMSCs with the tumor necrosis factor-related apoptosis-inducing ligand (TRAIL), exhibiting antitumor properties. After the in vitro coculture of the transduced GMSCs and TSCC cell lines, the necrosis and apoptosis of tumor cells were increased. In vivo studies revealed that transduced GMSCs injected to nude mice systemically inhibit TSCC cell growth, and after the selective engraftment into tumor tissues, through the mechanism of apoptosis [63].

IFNβ is known for its antitumor properties, such as apoptosis induction or tumor cell growth inhibition, through the JAK/STAT1 intracellular signaling pathway, as well as the activation of the host’s antitumor immune response [65]. The other types of MSCs transfected with IFNβ were already proven to exert anticancer properties, e.g., modified BM-MSCs-attenuated hepatocellular carcinoma, through inhibition by AKT/FOXO3a pathway [66], whereas the umbilical cord matrix-derived stem cells inhibited the growth of bronchioalveolar carcinoma by increasing apoptosis [67].

Du et al. [68] decided to undertake the same approach and transfected GMSCs with IFNβ. The authors observed that such modified GMSCs significantly inhibited TSCC cells proliferation in vitro and led to their apoptosis. After an intravenous injection into the mouse TSCC model, the transfected GMSCs selectively engrafted in TSCC xenograft, which resulted in smaller tumor volume, due to inhibition of tumor cell proliferation, and lower number of Ki-67-positive cells, which is highly expressed in various tumor tissues and used as a marker of tumor cell proliferation [68].

Coccè et al. [69] in vitro studies also revealed that GMSCs were able to uptake and release antineoplastic drugs, such as paclitaxel, doxorubicin, and gemcitabine, remaining less sensitive for their antiproliferative activity than cancer cells. The treatment of squamous carcinoma cells with the culture medium from drug-loaded GMSCs or their cell lysates resulted in dramatic inhibition of cancer cells growth, which was assessed via MTT assay.

Despite the lack of unanimity in indicating the role of MSCs in tumor promotion or suppression, it is significant that MSCs play a dynamic role within the tumor microenvironment. Further work is required to specify the intricate cross talk between MSCs and immune response at the tumor site.

### 5.2. Oral Mucositis

One of the major side effects of head and neck anticancer radio- and chemotherapy, affecting patients’ life quality, is inflammation of oral and GI tract mucosa. Similar symptoms are observed in patients with host-versus-graft disease. Oral mucositis represents challenging and painful symptoms characterized by atrophy, erythema, and ulceration that can lead to severe complications such as malnutrition and secondary infections.

Zhang et al. [34] demonstrated in an in vivo murine model of chemotherapy-induced oral mucositis that spheroid cultures derived GMSCs displayed improved cell plasticity and homing to mucositic lesions. Additionally, systemic infusion of GMSCs in experimental colitis in murine model has significantly improved histopathological severity of the colonic inflammation and clinically suppressed the overall disease activity.

### 5.3. Tissue Regeneration

The use of GMSCs seems to be particularly beneficial in various tissue regeneration, given gingival robust healing properties. Zhang et al. [51] reported systemic infusion of GMSCs into an excisional full-thickness skin wound splinting mouse model, which resulted in accelerated skin wound closure compared to the controls without the treatment. Rapid re-epithelialization, collagen deposition, and angiogenesis were also observed. GMSCs were hypothesized to promote skin wound healing by suppressing inflammatory cell infiltration and proinflammatory cytokine production, as well as increasing IL-10 production [51]. GMSCs were also used in tendon tissue regeneration in a study by Moshaverinia et al. [70]. Cells were encapsulated in TGF-β3-loaded RGD-coupled alginate microspheres and transplanted subcutaneously into immunocompromised mice, which resulted in wave-like aligned fibrils with tendon-like structure formation and ectopic neotendon regeneration as indicated by histological and immunohistochemical staining [70]. Ansari et al. [43] also encapsulated GMSCs in alginate microspheres loaded with myogenic differentiation cocktail and transplanted them subcutaneously into immunocompromised mice. Muscle-like structure formation were observed and histochemical, and immunofluorescent staining revealed the presence of myogenic cell-specific markers, such as Myosin II and MyoD, suggesting the possible use of GMSCs in muscle tissue regeneration [43].

Many studies aimed to utilize GMSCs in bone regeneration. Moshaverinia et al. [71], in another study, encapsulated GMSCs in a RGD-coupled alginate microspheres. Such encapsulated cells were transplanted into immunocompromised mice with 5-mm-diameter-critical-size calvarial defects, which resulted in bone fill. However, as indicated by microcomputed tomography analysis and histological staining, GMSCs were less efficient in bone regeneration than PDLSCs [71]. A systemic transplantation of GMSCs for bone regeneration was performed by Xu et al. [72] in C57BL/6J mice with defects in mandibular bone. The results revealed that GMSCs were able to home to the mandibular defect site and the bone regeneration was enhanced compared to the controls without the treatment [72]. Similar results were obtained by Wang et al. [73], who implanted GMSCs, seeded on type I collagen gel, into Sprague–Dawley rats with mandibular and calvarial defects. When coupled with poly-lactide (3D-PLA) scaffold, GMSCs or their conditioned medium exerted regenerative effect on calvarial defects in Wistar rats, as demonstrated by Diomede et al. [74]. GMSCs were also treated with an inhibitor of TGFβ signaling, which enhanced their osteogenic properties, resulting in new bone formation in minipig maxillary bone defect model [75].

GMSCs were also used for periodontal tissue regeneration. In a study by Sun et al. [76], GMSCs were transplanted systemically, through intravenous infusion, into mice with periodontitis. After the therapy, alveolar bone heights were increased and newly formed periodontal ligament and alveolar bone were reported [76]. Liu et al. [77] systemically transplanted GMSCs to hyperlipidemic mice with periodontitis, but they focused on their influence on lipid metabolism and inflammation. Besides the increased formation of the new bone and higher alveolar bone height, they observed a significant decrease in levels of triglyceride (TG), total cholesterol, low-density lipoprotein cholesterol (LDL), IL-6, TNFα, alveolar bone loss, and sterol regulatory element binding protein 1c (SREBP-1c). Moreover, the levels of high-density lipoprotein cholesterol (HDL), IL-10, and peroxisome proliferator-activated receptor α (PPARα) were increased, resulting in attenuated inflammation and hyperlipidemia [77]. Even the conditioned medium from GMSCs was shown to promote periodontal regeneration in rats, which was due to the regulation of inflammatory factors and enhancement of osteogenic differentiation of bone progenitor cells [78]. Therefore, it seems that soluble factors secreted by GMSCs are sufficient for stimulation of periodontal regeneration.

In several studies, GMSCs were used for nervous tissue regeneration. Zhang et al. [79] printed nerve constructs from GMSCs spheroids in the absence of exogenous scaffolds, using a scaffold-free 3D bioprinter system. Such constructs were transplanted to rats with segmental defects in facial nerves, which resulted in their enhanced regeneration and functional recovery [79]. Mammana et al. [80] utilized GMSCs for spinal cord injury treatment in mice. They pretreated the cells with nanostructured liposomes enriched with MOR (moringin) and administered them intravenously, which restored spinal cord normal morphology via COX2, GFAP, IL-1β, and IL-6 levels reduction. GMSCs also exerted aniapoptotic effects by decreasing levels of proapoptotic Bax, caspase 9, and caspase 3 [80]. Similar results were obtained by Rajan et al. [81], who utilized GMSCs-derived conditioned medium to treat scratch-injured murine motor neuron-like NSC-34 cells, which resulted in decreased levels of caspase 3, Bax, SOD-1 (superoxide dismutase 1), iNOS, and TNFα and increased levels of Bcl-2, IL-10, and neurotrophins, such as BDNF (brain-derived neurotrophic factor) and NT3 (neurotrophin 3). It was, therefore, hypothesized that conditioned medium from GMSCs may elicit neuroprotection and be useful in motor neuron injury treatment [81].

### 5.4. Immunologic- and Inflammatory-Related Disorders

Given the broad immunomodulatory properties of GMSCs, many authors have aimed to treat immunologic- and inflammatory-related disorders with the use of these cells in animal models. Murine contact hypersensitivity (CHS) is a model for human allergic contact dermatitis and both Su et al. [49] and Li et al. [82] demonstrated that systemic or local infusion of GMSCs mitigated CHS via decreased infiltration of inflammatory cells and suppression of inflammatory cytokines, which was due to the PGE2-dependent mechanisms. Rheumatoid arthritis is another condition, which could benefit from GMSCs therapy. After the infusion of GMSCs into collagen-induced arthritic mouse model, the severity of the disease decreased, i.e., the production of IFNγ and IL-17A was downregulated, whereas the number of CD4^+^CD39^+^FOXP3^+^ Treg cells increased, as indicated by Chen et al. [83]. Similar results were obtained by Gu et al. [84], who additionally revealed that such therapy induced T cell apoptosis via the FasL/Fas pathway, resulting in immune tolerance. Luo et al. [85], on the other hand, demonstrated that GMSCs transferred to collagen-induced arthritis mice suppress osteoclastogenesis and bone erosion, partly through CD39-adenosine signal pathway. The same signaling pathway was reported to be involved in GMSCs-mediated prevention of acute graft-versus-host-disease (GVHD) in mouse models, as indicated in studies by Huang et al. [86] and Ni et al. [87]. Immunomodulatory potential of GMSCs was also observed in the case of type I diabetes induced by streptozotocin in mice. After intraperitoneal injection, the levels of IL-17 and IFNγ decreased in CD4^+^ and CD8^+^ T cells, both in spleen and lymph nodes, which resulted in delayed diabetes onset and ameliorated pathology scores in pancreas, suggesting a possible novel therapy for this autoimmune disease [88].

## 6. Clinical Application in Stem Cell Therapies

The clinicaltrials.gov website was subject to the search terms “Gingival Mesenchymal Stem Cells,” “Gingival Stem Cells,” and “GMSC” to uncover clinical trials utilizing stem cells derived from gingival tissue. To date, only three clinical trials with GMSCs are listed on clinicaltrials.gov website, with one completed (NCT03638154), one active, not yet recruiting (NCT03570333), and one of unknown status (NCT03137979). The completed study (NCT03638154) was randomized, double-blind trial that enrolled 20 participants with intrabony periodontal defects. The aim of the study was to evaluate regenerative potential of GMSCs carried in β-tricalcium phosphate scaffold, and the bone gain was measured using radiographic imaging, yet no results were posted. The second mentioned study (NCT03570333) was a single-blind nonrandomized trial that aimed to compare the regenerative and differentiation potential of GMSCs isolated from premolar and molar region of the mouth within the same patient. The third study (NCT03137979) was triple-blind randomized trial, enrolling 30 participants with periodontitis. Basically, 10 patients received GMSCs seeded on collagen scaffolds after open flap debridement, and the aim was to test the safety and efficacy of such transplantation, via evaluation of alveolar bone regeneration and adverse reaction, among others.

## 7. Therapeutic Potential of GMSCs-Derived Exosomes

**Figure 4:**
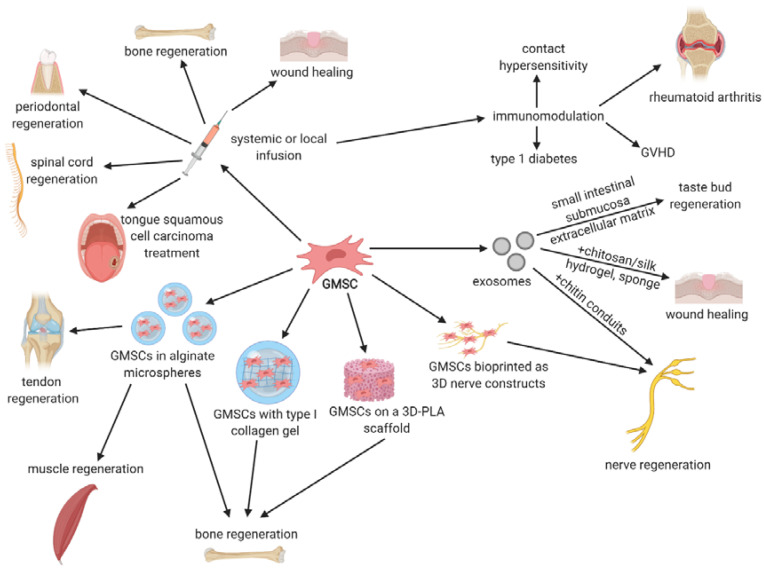
GMSCs may be injected, systemically or locally, or encapsulated in alginate microspheres, seeded on various types of scaffolds or gels, and bioprinted to facilitate bone, tendon, muscle, periodontal, nerve or spinal cord regeneration, wound healing, or tongue squamous cell carcinoma treatment, as well as in contact hypersensitivity, type 1 diabetes, graft-versus-host disease, or rheumatoid arthritis treatment. GMSCs-derived exosomes may be used for nerve or taste bud regeneration and wound healing. Created with BioRender.

Apart from GMSCs themselves, the GMSCs-derived exosomes may be used as a therapeutic tool. Exosomes are a subset of extracellular vesicles with a diameter of 40–160 nm, containing DNA, RNA, lipids, metabolites, and proteins, that originate from the invagination of endosomal membrane [89]. Since they have the ability to transfer their cargo to recipient cells, they are assumed to play an important part in cell-to-cell communication and may be used as a drug delivery system [90]. Exosomes are released by most mammalian cells, both in pathological and physiological conditions, therefore, their application as diagnostic biomarkers has been proposed [91]. In terms of GMSCs-derived exosomes’ therapeutic potential, only a few studies were conducted. In one of them, Rao et al. [92] reported their utilization in nerve regeneration, revealing that they were successful in promoting Schwann cell proliferation and axon growth in vitro. When combined with chitin conduits, these exosomes increased the number and diameter of nerve fibers and promoted myelin formation and motor function recovery in rats [92]. Shi et al. [93], on the other hand, combined exosomes from GMSCs with chitosan/silk hydrogel sponge and used them for treating wounds in a diabetic rat skin defect model. After the treatment, the deposition and remodeling of collagen and re-epithelialization occurred, and the microvessel and nerve density were increased compared to the control group [93]. In another study, GMSCs-derived exosomes were combined with small intestinal submucosa–extracellular matrix and transplanted into rats with a critical-sized tongue defect model, to examine the influence on taste bud regeneration [94]. Such therapy resulted in an increased expression of Shh, BDNF, CK14, CK8, and markers for type I, II, and II taste bud cells as compared to the controls, therefore facilitating taste bud regeneration and reinnervation, suggesting a possible application in tongue reconstruction [94]. The primary characteristics of exosomes, nanoscale size, membrane composition, stability and lack of toxicity, suggest innovative applications in clinical therapy. The possible clinical and preclinical application of GMSCs and their derivatives such as exosomes are presented schematically in the Figure 4.

## 8. Conclusions

The gingiva provides an alternative source of easily obtainable adult mesenchymal stem cells. They fulfill the minimal criteria for MSCs published by ISCT and are able to differentiate into cell lineages derived from all three primary germ layers in vitro, however in most cases, the in vivo differentiation was not confirmed. Their robust immunomodulatory potential was demonstrated both in vitro and in vivo and was exerted via direct cell-to-cell contact or soluble factor production, which indicates that not only GMSCs but also their conditioned media or their exosomes may be used in therapy. Indeed, such approach has been undertaken in several studies, providing promising results in terms of tissue regeneration and immunomodulation. GMSCs may also become a powerful tool in anticancer therapies, especially when engineered to express antitumor molecules. However, most of the studies utilizing GMSCs or their derivatives are at the preclinical stage. Although in many cases the results are promising, there is a need to conduct similar studies in humans to prove GMSCs’ safety and efficacy, as the appropriate dosage or long-term fate of transplanted GMSCs in the recipient remains unclear.

## Figures and Tables

**Table 1:** Overview of gingival mesenchymal stem cells (GMSCs) isolation and culture methods.

Isolation Method	Author	Publication Date	Composition of Culture Medium	Enzymes Used for Digestion	Time of Digestion
Explant method	El-Bialy et al. [24]	2014	DMEM, 10% FBS, 100 U/mL penicillin, 100 µg/mL streptomycin	–	–
	Fawzy El-Sayed et al. [23]	2015	α-MEM, 15% FCS, 400 mM/mL L-glutamine, 100 U/mL penicillin, 100 µg/mL streptomycin, 1% amphotericin	–	–
	Fournier et al. [25]	2010	DMEM, 20% FCS, 100 µg/mL penicillin, 100 µg/mL streptomycin, 2 ng/mL amphotericin	–	–
	Mitrano et al. [22]	2010	α-MEM, 10% FBS, 1% penicillin, streptomycin, amphotericin	–	–
Enzymatic method	Gao et al. [28]	2014	α-MEM, 10% FBS	4 mg/mL collagenase IV	2 h at 37 °C
	Ge et al. [31]	2012	α-MEM, 20% FCS, 2 mM L-glutamine, 100 µM L-ascorbate-2-phosphate, 1 mM sodium pyruvate, 50 U/mL penicillin, 50 µg/mL streptomycin, 2.5 µg/mL amphotericin	3 mg/mL collagenase I, 4 mg/mL Dispase II	50 min at 37 °C
	Jin et al. [32]	2015	α-MEM, 15% FBS, 100 U/mL penicillin, 100 µg/mL streptomycin, 200 mM L-glutamine, 10 mM ascorbic acid 2-phosphate	2 mg/mL collagenase IV, 1 mg/mL Dispase	30 at 37 °C (fraction discarded) 90 min at 37 °C (fraction seeded)
	Tang et al. [29]	2011	DMEM, 10% FBS, 0.292 mg/mL glutamine, 100 U/mL penicillin, 100 µg/mL streptomycin	0.66 mg/mL collagenase I	50 min at 37 °C
	Tomar et al. [30]	2010	α-MEM, 10% FCS	0.1% collagenase, 0.2% Dispase	15 min at 37 °C (fraction discarded) 5 min at 37 °C 10 min at 37 °C 15 min at 37 °C (fractions pooled and seeded)
	Zhang et al. [17]	2009	α-MEM, 10% FBS, 100 U/mL penicillin, 100 µg/mL streptomycin, 2 mM L-glutamine, 100 mM nonessential amino acids	4 mg/mL collagenase IV	2 h at 37 °C

**Table 2:** Overview of published studies utilizing GMSCs in anticancer therapies.

Author	Publication Date	Type of Cancer	Employed Method	Obtained Results
Coccè et al. [69]	2017	TSCC line (SCC154)	Drug loading of GMSCs with paclitaxel, doxorubicin, and gemcitabine	Growth inhibition of cancer cells in response to drug release by GMSCs
Du et al. [68]	2019	TSCC line (CAL27); TSCC xenograft model in BALB/c nude mice	Transfection of GMSCs with IFNβ	Growth inhibition and apoptosis of cancer cells in vitro and inhibition of tumor cell proliferation in vivo
Ji et al. [62]	2016	TSCC lines (CAL27, WSU-HN6)	Direct and Transwell coculture of GMSCs and cancer cells; simultaneous subcutaneous injection of cancer cells with GMSCs	Growth inhibition of cancer cells in vitro and in vivo
Xia et al. [63]	2014	TSCC line (TCA8113, CAL27)	Transfection of GMSCs with TRAIL	Tumor cell necrosis and apoptosis in vitro and in vivo
